# Case Report: The Role of Molecular Analysis of the *MUTYH* Gene in Asymptomatic Individuals

**DOI:** 10.3389/fgene.2020.590486

**Published:** 2020-12-15

**Authors:** Katarína Fabišíková, Olívia Hamidová, Regína Lohajová Behulová, Katarína Závodná, Petra Priščáková, Vanda Repiská

**Affiliations:** ^1^ Faculty of Medicine, Institute of Medical Biology, Genetics and Clinical Genetics, Comenius University, Bratislava, Slovakia; ^2^ Department of Clinical Genetics, St. Elizabeth Cancer Institute, Bratislava, Slovakia

**Keywords:** case report, MUTYH glycosylase, *MUTYH* gene, compound heterozygote, germline mutation, pathogenic variant, base excision repair

## Abstract

*MUTYH*-associated polyposis (MAP) is a rare hereditary condition caused by the biallelic mutation in the *MUTYH* gene encoding MUTYH glycosylase. This enzyme is a key member of the base excision repair (BER) pathway responsible for the repair of DNA lesions formed by reactive oxygen species (ROS). We report two cases of MAP. In case 1, a 67-year-old woman who presented with a personal history of colorectal and endometrial cancer and a family history of cancer syndromes underwent multigene panel testing that revealed a germline homozygous (biallelic) pathogenic variant c.1187G > A (p.Gly396Asp) in the *MUTYH* gene. Subsequent sequencing analysis performed in the offspring of the proband identified all three asymptomatic offspring as carriers of this pathogenic variant. In case 2, a 40-year-old woman with a strong family history of colorectal cancer [the proband’s sister was a carrier of the pathogenic variant c.536A > G (p.Tyr179Cys) of the *MUTYH* gene] and renal cancer underwent sequencing analysis of the *MUTYH* gene. The pathogenic heterozygous (monoallelic) variant c.536A > G (p.Tyr179Cys) of the *MUTYH* gene was identified in the proband. We found another pathogenic variant of the *MUTYH* gene—heterozygous (monoallelic) mutation c.1187G > A (p.Gly396Asp) in the genome of the proband’s husband. Molecular analysis of their offspring revealed that they are compound heterozygotes for *MUTYH* pathogenic variants c.536A > G (p.Tyr179Cys)/c.1187G > A (p.Gly396Asp). This paper shows the importance of genetic testing of asymptomatic relatives of the proband to ensure an early surveillance and management of individuals positive for pathogenic variant (s) in the *MUTYH* gene.

## Introduction

Hereditary colorectal cancer (CRC) is mostly caused by mutations in the *APC* (adenomatous polyposis coli) gene or mismatch repair genes (*MLH1*, *MSH2*, *MSH3*, *MSH6*, *PMS2*, and *EPCAM2*).

In 2002, Al-Tassan et al. first described inherited variants in the *MUTYH* gene that led to an increased number of somatic inactivating mutations in the *APC* gene. Most of these somatic mutations were G:C to T:A transversions as a result of the impaired (defective) base excision repair (BER; Al-Tassan et al., 2002). The BER pathway, involving the DNA repair glycosylase MUTYH, repairs DNA lesions formed by reactive oxygen species (ROS).

ROS are natural by-products of normal aerobic metabolism. Elevated production of ROS arises from cellular exposure to toxins, ultraviolet light, and ionizing radiation. 8-Oxo-7,8-dihydroguanine (8-oxoG) is the most common, stable, and mutagenic oxidatively damaged guanine lesion (Banda et al., 2017). This nucleotide mispairs with adenine and MUTYH glycosylase removes these mispaired adenines (Terdiman, 2009).

*MUTYH*-associated polyposis (MAP) is an autosomal recessive disorder that can be diagnosed in patients with an attenuated colonic polyposis phenotype (Kidambi et al., 2018). Detection of germline *MUTYH* mutations is recommended in individuals affected by multiple colorectal adenomas in whom no mutation in the *APC* gene had been identified (Isidro et al., 2004). MAP is caused by biallelic pathogenic variants in the *MUTYH* gene and is characterized by the presence of 15–100 colorectal polyps and an increased carrier’s risk of colorectal adenomas and carcinomas. Patients are diagnosed with MAP at a mean age of 45 years. The chance of developing CRC reaches 20–80% in patients aged from 50 to 80 years (Lubbe et al., 2009; Castillejo et al., 2014). MAP is estimated to account for 0.7% of all CRC and between 0.5 and 6% of cohorts of familial or early-onset CRC in which affected individuals have a low number of adenomas (<15–20) (Sieber et al., 2003; Cleary et al., 2009; Lubbe et al., 2009; Landon et al., 2015; Pearlman et al., 2017). Seventy percent of *MUTYH* mutations involve c.1187G > A (p.Gly396Asp) and c.536A > G (p.Tyr179Cys) mutations (Sampson and Jones, 2009).

*MUTYH* mutations can contribute to the development of sporadic gastric cancer. The presence of *MUTYH* pathogenic variants is an independent predictor of poor prognosis in sporadic gastric cancer and in salivary gland secretory carcinoma, while its inhibition has been shown to reduce the survival of pancreatic ductal adenocarcinoma cells (Curia et al., 2020).

Biallelic (compound heterozygous or homozygous) *MUTYH* mutations inherited from both parents occur in 0.01–0.04% of the Caucasian population and are associated with an 18- to 100-fold increased risk of CRC compared with the general population. Carriers of biallelic *MUTYH* mutation have an increased risk of extracolonic cancers such as ovarian cancer, urinary bladder cancer, cancer of the upper gastrointestinal tracts, breast cancer, endometrial cancer, and skin cancer compared with the general population (Win et al., 2016; Table 1).

Monoallelic (heterozygous) *MUTYH* mutations, inherited from only one parent, occur in 1–2% of the Caucasian population and are associated with a moderately increased risk of CRC (Win et al., 2016). Carriers of monoallelic mutation have on average an approximately 2.5-fold increased risk of CRC compared with the general population (Win et al., 2014). It is estimated that there are an elevated risk of liver and gastric cancers and a slightly increased risk of breast cancer for carriers with monoallelic *MUTYH* mutation (Zhu et al., 2011; Rennert et al., 2012).

It is appropriate to perform molecular genetic analysis of asymptomatic relatives for the *MUTYH* pathogenic variants identified in the proband to ensure appropriate surveillance (beginning at the age of 10–15 years) and early identification of polyps (Nielsen et al., 2012, updated 2019).

## Case Description

### Case 1 Description

We performed multigene panel testing of the patient with a family history of cancer syndromes. The proband was a 67-year-old woman presenting with a personal history of CRC diagnosed at the age of 54 and endometrial cancer diagnosed at the age of 65. Immunohistochemistry (IHC) demonstrated intact expression of *MLH1*, *MSH2*, *MSH6*, and *PMS2*. Colorectal tumor that developed was microsatellite stable.

**Figure 1:**
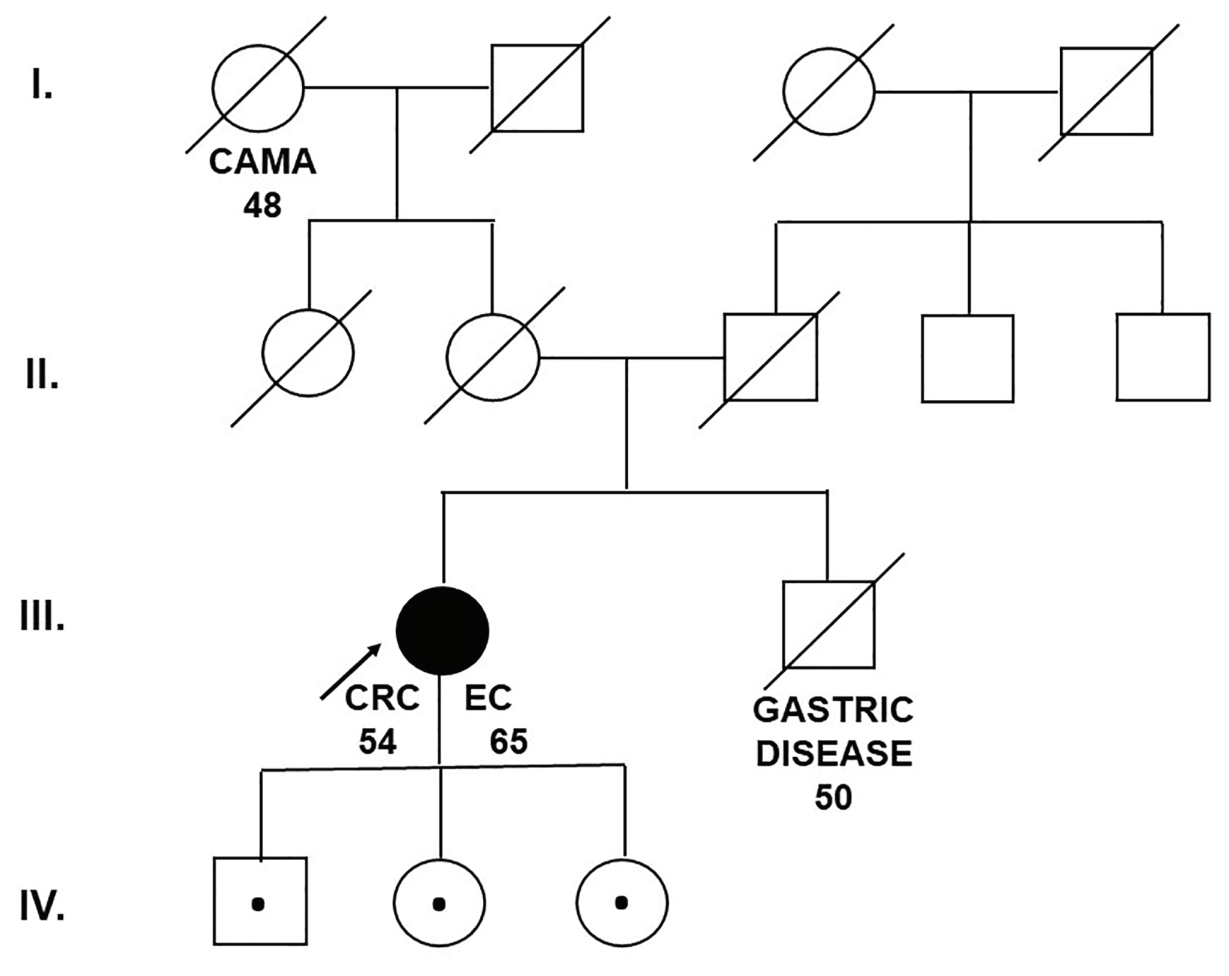
Pedigree of the family carrying the MUTYH mutation c.1187G>A (p.Gly396Asp). CAMA, breast cancer; CRC, colorectal cancer; EC, endometrial cancer (Department of Clinical Genetics, St. Elizabeth Cancer Institute, Bratislava, Slovakia).

The grandmother of the patient developed breast cancer at the age of 48. The brother of the proband died aged 50 from an unknown gastrointestinal disease (probably gastric cancer; Figure 1).

**Table 1:** Cancer risks in individuals with MUTYH polyposis compared with the general population (Nielsen et al., 2012, updated 2019).

Cancer type	General population risk^1^	Risk associated with MAP^2^	Median age of onset
Colorectal	5.5%	43–63% by the age 60 years; 80–90% lifetime risk without surveillance	48 years
Duodenal	<0.3%	4%	61 years
Ovarian	1.3%	6–14%	51 years
Bladder	1–4%	6–8% in females; 6–25% in males	61 years
Breast	12%	12–25%	53 years
Endometrial	2.9%	~3%	51 years
Gastric	<0.7–1%	1%	38 years
Pancreatic	1.6%	Unclear if the risk for this type of cancer is increased in individuals with MAP	-
Skin	~20%^3^	Unclear if the risk for this type of cancer is increased in individuals with MAP	-
Thyroid	0.6–1.8%	Unclear if the risk for this type of cancer is increased in individuals with MAP	-

1US National Cancer Institute’s Surveillance Epidemiology and End Results (SEER) Database 2012–2014.2Nielsen et al. (2006), Lubbe et al. (2009), Vogt et al. (2009), Win et al. (2014), and Walton et al. (2016).3Unclear if the risk for this type of cancer is increased in individuals with MAP.

A panel of 26 cancer predisposition genes identified a germline homozygous (biallelic) pathogenic variant c.1187G > A (p.Gly396Asp) in exon 13 of the *MUTYH* gene in the proband.

The c.1187G > A (p.Gly396Asp) variant is a common cause of MAP, and this missense variant disrupts MUTYH protein function. Furthermore, panel testing revealed also a heterozygous variant of the *PALB2* gene [c.229 T > C (p.Cys77Arg)] and a heterozygous variant of the *TP53* gene (c.1100 + 30A > T). However, according to the ACMG guidelines for the interpretation of sequence variants, both variants are of uncertain significance (VUS); thus, we suggest that they have no significant impact on the proband’s cancer etiology.

*MUTYH* molecular genetic testing was offered to the husband of the proband to determine the risk of MAP in the offspring, but he did not agree to being tested.

Sequencing analysis of the whole coding sequence of the *MUTYH* gene was subsequently conducted in the offspring of the proband: one son (aged 38) and two daughters (aged 47 and 39). The germline heterozygous (monoallelic) *MUTYH* mutation c.1187G > A (p.Gly396Asp) was found in all three asymptomatic offspring of the proband.

### Case 2 Description

**Figure 2:**
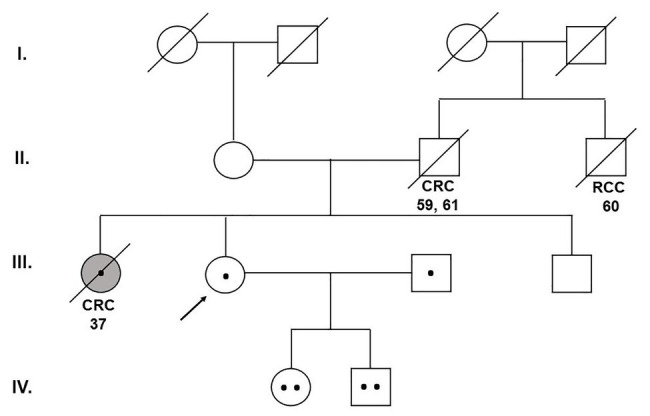
Pedigree of the family carrying the MUTYH mutations c.1187G>A (p.Gly396Asp) and c.536A>G (p.Tyr179Cys). CRC, colorectal cancer; RCC, renal cell cancer (Department of Clinical Genetics, St. Elizabeth Cancer Institute, Bratislava, Slovakia).

A 40-year-old woman was presented to the Department of Clinical Genetics for genetic counseling due to a strong family history of CRC and renal cell carcinoma (RCC). The proband’s sister was a carrier of the germline pathogenic heterozygous (monoallelic) mutation c.536A > G (p.Tyr179Cys) in exon 7 of the *MUTYH* gene (the result of the 26-gene panel examination) and she died from CRC at the age of 37. The proband’s father developed CRC at the age of 59. At the age of 61, he died after the recurrence of CRC. The brother of the proband’s father died from RCC at the age of 60 (Figure 2).

The proband underwent sequencing analysis of exon 7 for detection of the presence of *MUTYH* pathogenic variant c.536A > G (p.Tyr179Cys) found in her sister with a positive result—carrier of monoallelic *MUTYH* pathogenic variant. To determine the risk of MAP in the offspring, we performed sequencing analysis of the whole *MUTYH* gene also in the proband’s husband. We identified the pathogenic heterozygous (monoallelic) mutation c.1187G > A (p.Gly396Asp) in exon 13 of the *MUTYH* gene in the husband of the proband.

The proband’s asymptomatic offspring—one son aged 11 and one daughter aged 18—subsequently underwent genetic counseling and sequencing analysis. We identified that both son and daughter of the proband are carriers of germline pathogenic variants c.536A > G (p.Tyr179Cys; passed down to offspring from the mother carrying a mutation) and c.1187G > A (p.Gly396Asp; passed down to offspring from the father carrying a mutation) of the *MUTYH* gene. In this way, the offspring of the proband are combined heterozygotes and this genotype refers to the diagnosis of MAP requiring patient’s management.

## Discussion

These two cases demonstrate the importance of genetic testing of asymptomatic partners and the proband or carrier offspring of the pathogenic variant. We identified in our probands and their relatives pathogenic variants in the *MUTYH* gene by next-generation sequencing (NGS) panel testing and Sanger sequencing.

Variants c.1187G > A (p.Gly396Asp) and c.536A > G (p.Tyr179Cys) are the two most common pathogenic variants in the *MUTYH* gene responsible for the MAP. MAP is associated with an increased lifetime risk of CRC development. Other features involve thyroid nodules, benign adrenal lesions, jawbone cysts, and congenital hypertrophy of the retinal pigment epithelium (Nielsen et al., 2012, updated 2019). The risk of CRC is strongly age dependent, with incomplete penetrance at the age of 60 (Lubbe et al., 2009). Several studies found the association between *MUTYH* variants and the risk of breast cancer. In a case-control study, 930 women with a high prevalence of *MUTYH* mutations were investigated for the two variants c.1187G > A (p.Gly396Asp) and c.536A > G (p.Tyr179Cys), and patients with breast cancer revealed a 6.7% prevalence of c.1187G > A (p.Gly396Asp) (Rennert et al., 2012).

Recently, it has been estimated that *MUTYH* mutations could cause some diseases that are not associated with polyposis, such as Parkinson’s disease, Alzheimer’s disease, Friedreich’s ataxia, Huntington’s disease, retinitis pigmentosa, and neurofibromatosis (Curia et al., 2020).

Several functional assays have shown that the MUTYH glycosylase activity is greatly reduced for the *MUTYH* pathogenic variant c.536A > G (p.Tyr179Cys) (a 98% reduction of glycosylase activity) compared with the *MUTYH* pathogenic variant c.1187G > A (p.Gly396Asp) (an 86% reduction of glycosylase activity) (Al-Tassan et al., 2002; Nielsen et al., 2009; Banda et al., 2017). MAP patients harboring homozygous *MUTYH* pathogenic variant c.536A > G (p.Tyr179Cys) showed more severe clinical features than those with homozygous *MUTYH* pathogenic variant c.1187G > A (p.Gly396Asp). Furthermore, patients with a homozygous c.1187G > A (p.Gly396Asp) mutation or compound heterozygous c.1187G > A (p.Gly396Asp)/c.536A > G (p.Tyr179Cys) mutations have a lower risk of CRC development as compared with patients with a homozygous c.536A > G (p.Tyr179Cys) mutation.

According to the functional studies mentioned above, the mean age of CRC diagnosis is 46 years for the homozygous c.536A > G (p.Tyr179Cys) mutation, 52 years for the heterozygous c.1187G > A (p.Gly396Asp)/c.536A > G (p.Tyr179Cys) mutation, and 58 years for the homozygous c.1187G > A (p.Gly396Asp) mutation.

It is suitable to begin surveillance earlier for p.Tyr179Cys homozygotes than for p.Gly396Asp homozygotes and p.Gly396Asp/p.Tyr179Cys compound heterozygotes (Nielsen et al., 2011).

According to the NCCN guidelines (2019), the management of monoallelic *MUTYH* pathogenic variant carriers involved undergoing colonoscopy every 5 years beginning at the age of 40 years. The identification of monoallelic carriers of *MUTYH* pathogenic variants is very important to select individuals who might benefit from preventive strategies.

It is appropriate to clarify the genetic status of apparently asymptomatic individuals to reduce morbidity and mortality in those who would benefit from appropriate surveillance (beginning at the age of 10–15 years) and early identification and treatment of polyps. That is why we recommended for both children to undergo colonoscopy and upper endoscopy (the frequency of colonoscopy is recommended every 1–2 years starting at the age of 25 in case of a negative result, the frequency of upper endoscopy every 3–4 years starting at the age of 30). We also recommended thyroid ultrasound and skin examination by a dermatologist for both children. For girls, we recommended regular gynecologic and mammalogical dispensarization starting at the 21st year.

Identification of mutations in cancer predisposition genes is a challenge for current cancer risk management, counseling, and treatment decision-making regarding patients and their families.

## Conclusion

Polyposis syndromes are not only due to mutations in well-known and well-explored cancer predisposition genes. Here, we suggest the need for the extension of molecular analysis for genes that are mutated with a relatively low frequency but with a strong impact on CRC development. Mutations in the *MUTYH* gene are associated with an 18- to 100-fold increased risk of CRC and an elevated risk of extracolonic cancers in comparison with the general population (Win et al., 2016).

Genetic counseling and testing with a multigene panel could be considered for all patients with a personal or family history of cancer syndromes. Genetic screening can provide early diagnosis and improve prognosis.

Potential future implementation of the exome sequencing indicates the discovery and investigation of new candidate genes involved in the etiology of the CRC.

## Data Availability Statement

The raw data supporting the conclusions of this article will be made available by the authors, without undue reservation.

## Ethics Statement

Ethical approval was not provided for this study on human participants because this research was performed as a part of a routine molecular diagnostic of patients. Written informed consent to participate in this study was provided by the participants’ legal guardian/next of kin. Written informed consent was obtained from the individual(s), and minor(s) legal guardian/next of kin, for the publication of any potentially identifiable images or data included in this article.

## Author Contributions

All authors listed have made substantial, direct and intellectual contribution to the work and approved it for publication.

### Conflict of Interest

The authors declare that the research was conducted in the absence of any commercial or financial relationships that could be construed as a potential conflict of interest.
